# Rabies virus circulation in domestic mammals from a peri-urban area at the Pedra Branca Forest, Rio de Janeiro, Brazil

**DOI:** 10.1590/0037-8682-0228-2026

**Published:** 2026-09-25

**Authors:** Natasha Avila Bertocchi, Ellen Schmidt, Roberto Leonan Morim Novaes, Ademar Luiz Gomes do Couto, Jaíne Gonçalves Garcia, Helena Beatriz de Carvalho Ruthner Batista, Ricardo Moratelli

**Affiliations:** 1 Fundação Oswaldo Cruz, Fiocruz Mata Atlântica, Rio de Janeiro, RJ, Brasil.; 2 Instituto Pasteur de São Paulo, Seção de Diagnóstico da Raiva, São Paulo, SP, Brasil.

Dear Editor: 

The presence of anti-rabies virus (RABV) antibodies was investigated in farm animals from a peri-urban area at the Pedra Branca Forest, in the state of Rio de Janeiro, Brazil using the Rapid Fluorescent Focus Inhibition Test (RFFIT). A donkey without clinical signs or a vaccination history tested seropositive (≥0.5 IU/mL), indicating RABV exposure. This finding suggests the circulation of RABV in this area and corroborates previous evidence of anti-RABV antibodies detected in local bat populations. These results highlight the need for continuous rabies surveillance in domestic animals in peri-urban regions, where the interface between wildlife, domestic animals, and humans may facilitate viral transmission between species.

Rabies is a viral zoonotic disease of public health importance. It can affect all mammal species in all environments. RABV is a member of the genus *Lyssavirus* (*Rhabdoviridae*) and is transmitted primarily through the bite of infected mammals. Although cattle are the main animals affected by rabies transmitted by the common vampire bat (*Desmodus rotundus*) in Brazil, other herbivores such as horses, donkeys, and goats may also be affected due to extensive animal husbandry across the country^1^. These farm animals may also come into contact with the virus in peri-urban areas. The detection of anti-RABV antibodies in unvaccinated animals, thus, is an important indicator of viral circulation and suggests a potential risk or transmission^2^ ^,^ ^3^. 

The Pedra Branca Forest is the largest urban forest remnant in the world, and is an important biodiversity refuge within the metropolitan area of the state of Rio de Janeiro. This forest includes a variety of habitat types, which are subject to different anthropogenic impacts^4^. In addition to the expansion of residential occupation, other forms of human pressure, such as small-scale agricultural activities, which often keep domestic animals to assist with property maintenance tasks, are also present. This study was conducted in March 2024 on a smallholding adjacent to the Piraquara headquarters of Pedra Branca State Park (Parque Estadual da Pedra Branca), Rio de Janeiro State, Brazil **(**22°54′03″S, 43°26′26″W; Figure 1A, B **).** Samples were obtained from domestic animals raised on the smallholding that were unvaccinated against rabies. *D. rotundus* occurs in this locality and feeding interactions between *D. rotundus* and domestic animals have been reported^5^. 

**FIGURE 1:**
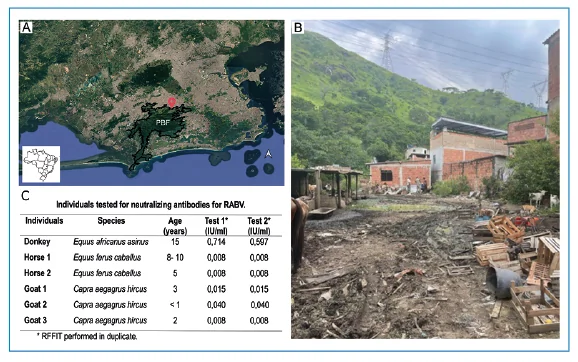
Sampling location and rabies virus (RABV) antibody testing results. **(A)** Map of the state of Rio de Janeiro, indicating the location of Pedra Branca Forest (PBF), and the sampling site in Piraquara (red marker). **(B)** Smallholding where blood samples were collected from domestic animals. **(C)** Rapid Fluorescent Focus Inhibition Test (RFFIT) results showing a donkey with a neutralizing antibody titer >0.5 IU/mL on two separate tests, indicating RABV exposure.

Blood samples were collected from six farm animals (2 horses, 3 goats, and 1 donkey) via jugular venipuncture. The samples were centrifuged to separate the serum, which was stored at −80°C until testing. To investigate the presence of anti-RABV antibodies, serum samples were tested using the RFFIT^6^. Briefly, the detection of neutralizing antibodies against RABV was performed using the Challenge Virus Standard (CVS) strain and Baby Hamster Kidney (BHK-21) cells at a concentration of 2.5 × 10⁴ cells/well. The neutralizing antibody titer was then calculated using the Reed and Muench method. Serum samples with antibody titers ≥0.5 IU/mL were considered positive^7^. Each sample was tested in duplicate using the RFFIT assay. Of the six animals tested, only the donkey (*Equus africanus asinus*) tested positive for anti-RABV antibodies (Figure 1C).

This finding indicates RABV circulation in domestic animals in the surrounds of Pedra Branca Forest. Rabies cases in domestic herbivores in Brazil occur predominantly in cattle, due to the extensive livestock production in the country, and approximately 99% of cases occur as a result of RABV transmission by *D. rotundus* ^1^. Unlike cattle, equines, and especially donkeys, are often used by smallholders in peri-urban areas for both urban and rural labor, as at the study site. Consequently, the potential role of this group of domestic animals in maintaining the local rabies transmission cycle may be overlooked^8^. Twelve rabies cases were reported in cattle and horses in the Pedra Branca Forest region in 2011 and 2012^9^. Furthermore, Cocchi *et al*.^3^ detected anti-RABV antibodies in 5 of 60 bats (8%) from the family Phyllostomidae, including *D. rotundus*, sampled in the Pedra Branca Forest in 2024, highlighting the importance of continuous serosurveillance in these animals of anti-RABV antibodies in bats in the metropolitan region of Rio de Janeiro.

The RFFIT is globally recognized as the standard assay for detecting anti-RABV neutralizing antibodies. The World Health Organization (WHO) considers serum samples with RABV antibody titers ≥0.5 IU/mL as positive in both humans and vaccinated domestic animals^7^. However, in wild animals without a history of prior vaccination, antibody titers ≥0.1 IU/mL may be considered positive^10^. Among the six domestic animals tested using the RFFIT, only the donkey tested positive. This finding suggests that the animal was exposed to RABV but did not develop the disease. In regions where rabies is considered endemic, anti-RABV antibodies have been detected in healthy, unvaccinated domestic (*e.g*., dogs) and wild animals, indicating active environmental RABV circulation^2^.

According to an interview with the farm owner, the farm animals had never been vaccinated, and the last public vaccination campaign in the area had taken place more than 10 years previously. Regarding rabies immunity, the Brazilian Ministry of Agriculture, Livestock and Supply (MAPA) states that domestic herbivores retain vaccine-induced immunity for a maximum of 12 months^11^. Information on rabies in donkeys is extremely limited or virtually nonexistent. However, in previously unvaccinated horses, the primary immune response to rabies vaccination leads to antibody titers below 0.5 IU/mL, and titers decline within a few weeks after the second dose of vaccine in older animals^12^. Considering the lack of recent vaccination campaigns and the short duration of immunity provided by rabies vaccines in domestic herbivores, the finding of a donkey testing seropositive for anti-RABV in an area where RABV is circulating in hematophagous bats^3^, highlights the importance of maintaining continuous serosurveillance and epidemiological surveillance in peri-urban regions surrounding the Pedra Branca Forest in Rio de Janeiro, as exposure to rabies in domestic animals within these areas may facilitate viral maintenance and increase the risk of transmission to humans. Furthermore, many people in the Pedra Branca peri-urban region are socio-economically vulnerable with limited access to veterinary care and public health information. Thus, integrating strategies combining continuous surveillance, health education, and sustained vaccination programs, are essential to mitigate the risk of rabies emergence.
